# Antiartherosclerotic Effects of Plant Flavonoids

**DOI:** 10.1155/2014/480258

**Published:** 2014-05-27

**Authors:** Shamala Salvamani, Baskaran Gunasekaran, Noor Azmi Shaharuddin, Siti Aqlima Ahmad, Mohd Yunus Shukor

**Affiliations:** Department of Biochemistry, Faculty of Biotechnology and Biomolecular Sciences, Universiti Putra Malaysia, 43400 Serdang, Selangor, Malaysia

## Abstract

Atherosclerosis is the process of hardening and narrowing the arteries. Atherosclerosis is generally associated with cardiovascular diseases such as strokes, heart attacks, and peripheral vascular diseases. Since the usage of the synthetic drug, statins, leads to various side effects, the plants flavonoids with antiartherosclerotic activity gained much attention and were proven to reduce the risk of atherosclerosis *in vitro* and *in vivo* based on different animal models. The flavonoids compounds also exhibit lipid lowering effects and anti-inflammatory and antiatherogenic properties. The future development of flavonoids-based drugs is believed to provide significant effects on atherosclerosis and its related diseases. This paper discusses the antiatherosclerotic effects of selected plant flavonoids such as quercetin, kaempferol, myricetin, rutin, naringenin, catechin, fisetin, and gossypetin.

## 1. Introduction

Being a chronic inflammatory disease, atherosclerosis is becoming the leading cause of death in most of the developed countries [1]. Cardiovascular diseases (CVDs) like myocardial infarction (heart attack), acute coronary syndrome, or stroke arise through the development of plaques and lesions inside the arteries [2–5]. Hypercholesterolemia, hypertension, and obesity give high risks for the progression of CVDs. Statins are widely used as the clinical treatment for atherosclerosis due to its excellent efficacy in reducing the low density lipoprotein (LDL) level [6, 7]. Statins competitively inhibit the HMG-CoA reductase enzyme that plays a great role in catalyzing the rate-limiting step in the biosynthesis of cholesterol [8]. The increase in hepatic LDL receptors' expression is triggered by the reduction of hepatocyte cholesterol concentration and helps to clear LDL from the circulation [9, 10].

However, the consumption of statins causes adverse health effects such as liver injury and muscle toxicity [10, 11]. The other side effects include myopathy, rhabdomyolysis, and acute renal failure [12]. Thus, attention is now directed to the natural products from plant origin that possess antiartherosclerotic activity and can promote human health. This can eventually avoid possible health effects due to the long period consumption of statins. Many researches on bioactive compounds and their possible medicinal attributes have been studied during the past decades [13–15]. Plant and plant by-products can be used for isolating health-promoting bioactive compounds since there are substantial plant sources which are relatively inexpensive. The bioactive compound from plant extracts has shown plentiful health-promoting effects in both* in vitro* and* in vivo* studies, such as antioxidant [16, 17], hypoglycemic [18–20], hypotensive [21], and hypocholesterolemic [22–24] effects. The aim of this review is to provide the reader with some important evidence on the antiatherosclerotic activity of selected flavonoids that are mostly found in plants.

## 2. Flavonoids

Flavonoids represent a broad family of more than 4000 secondary plant metabolites. The four predominant classes are 4-oxoflavonoids (flavones and flavonols), isoflavones, anthocyanins, and flavan-3-ol derivatives (tannins and catechin) [25–27]. For centuries, preparations that contain flavonoids are applied as the primary physiologically active components that have been used for treating human diseases [28]. Epidemiological studies have shown that the risk of heart diseases can be reduced through the consumption of flavonoid-rich diets [29]. Flavonoids may inhibit the vascular diseases' development through alteration in endothelial cell eicosanoid production [30]. Flavonoids also showed blood pressure lowering effect in hypertensive and normotensive subjects while flavonoids may have beneficial actions in obesity due to their capacity to regulate fatty oxidation and improve adipocyte functionality [31]. Besides, food derived flavonols (quercetin, kaempferol, and myricetin) have been reported to exhibit various biological functions and medicinal properties such as antioxidant, antithrombotic, anti-inflammatory, anti atherogenic, antiatherosclerotic, and cardioprotective effects [32–35]. The plants like* Garcinia cambogia* [36],* Mangifera indica* [37],* Hypericum perforatum* L [38], and* Asparagus racemosus* [39] that contain flavonoids have been proven to significantly lower the risk of atherosclerosis and CVD.

### 2.1. Quercetin

Flavonoids such as quercetin (3′,4′,3,5,7-pentahydroxyflavone) have gained considerable attention mainly due to their broad spectrum of health beneficial effects for the treatment of CVDs. Quercetin has been reported to improve endothelium-dependent vasorelaxation in aorta, decreases systolic blood pressure, and reduces cardiac hypertrophy and proteinuria in hypertensive rats [40, 41]. Sánchez et al. [42] reported that enhancement of endothelial nitric oxide synthase (eNOS) activity and reduction of nicotinamide adenine dinucleotide phosphate (NADPH) oxidase-mediated superoxide production with downregulation of p47^phox^ expression showed the antihypertensive effects of quercetin. Besides that, quercetin has been proven to improve dyslipidemia, decrease oxidative stress through stimulation of lipolysis activity, and upregulate the adipocytes genes expression which increases the lipids beta oxidation [43, 44]. Quercetin treatment in obesity animal models showed reduction in body weight, visceral and subcutaneous adipose tissue, and liver fat accumulation. Moreover, quercetin also suppressed the peroxisome proliferator-activated receptor y (PPARy) and sterol regulatory element-binding proteins (SREBP) expression. The reduction in the expression of PPARy indicates reduction in adipogenesis [45–47]. On the other hand,* Morus alba* L leaves containing quercetin 3-(6-malonylglucoside) (Q3MG) as their major flavonol attenuated the development of artherosclerotic lesion in LDL receptor-deficient mice through LDL resistance enhancement to oxidative modification and the artherosclerotic lesion in* M. alba*-treated mice was significantly reduced by 52% [48]. Kleemann et al. [35] reported on the anti-inflammatory and antiatherogenic effects of the quercetin and have shown that short-term treatment for 14 days with dietary quercetin managed to completely quench the cytokine-induced expression of human C-reactive protein (CRP) in transgenic mice. The elevating level of CRP is an inflammation marker that increases the risk of CVDs [35, 49]. Bhaskar et al. [50] investigated the antiatherosclerotic property of quercetin and found notable regression of atherosclerosis in the histopathological examination of the aorta in hypercholesterolemic rabbits supplemented with quercetin. This suggests the potential of quercetin as an alternative therapeutic agent for atherosclerosis and CVDs as well as for hypertension and obesity that can lead to CVDs [50–52].* Camellia chinensis* [53, 54],* Allium fistulosum* and* Calamus scipionum *[54],* Moringa oleifera* [17, 55],* Centella asiatica *[56],* Hypericum hircinum* [57], and* Hypericum perforatum* [58] have been reported to have high content of quercetin.

### 2.2. Kaempferol

Numerous researches have been conducted on kaempferol (3,4′,5,7-tetrahydroxyflavone) and studies have shown that consumption of kaempferol-rich foods reduced the risk of developing cardiovascular diseases [59, 60]. Kaempferol has been reported to increase endothelium relaxation in coronary artery of porcine [61]. Xiao et al. [62] investigated kaempferol's protective effects against endothelial damage and found that kaempferol improves the nitric acid production and reduces asymmetric dimethylarginine level which enhances the endothelium-dependent vasorelaxation, preventing endothelium injuries and oxidative damage in cells. The ability of kaempferol in reducing oxidative stress can be the beneficial effect in CDVs [63]. Kaempferol also prevents arteriosclerosis by the inhibition of LDL oxidation and formation of platelets. Kowalski et al. [64] demonstrated that the monocyte chemoattractant protein (MCP-1) is inhibited by kaempferol in an* in vitro* study. MCP-1 involves in the initial stage of plaque formation in arteriosclerosis. Kong et al. [65] evaluated the effect of kaempferol on atherosclerosis induced rabbit models, and upon 10-week treatment of kaempferol with high cholesterol diet, the expression of intercellular adhesion molecule-1 (ICAM-1), vascular adhesion molecule-1 (VCAM-1), and MCP-1 in the rabbits' aorta has been significantly downregulated. This indicates that kaempferol can alleviate vascular inflammation to prevent atherosclerosis.* Moringa oleifera* constitutes kaempferol as one of its major bioactive compound [17, 55] which was proven to possess antiatherosclerotic and hypolipidemic properties and has therapeutic potential in the treatment of hyperlipidemia, atherosclerosis, and cardiovascular diseases [66, 67]. Therefore, kaempferol can be considered to be an effective and potent agent against atherosclerosis. The presence of kaempferol has been identified in many other plants and some of them are* Centella asiatica* [56],* Euonymus alatus* [68],* Kaempferia galanga* L [69],* Ginkgo biloba*,* Equisetum spp.*,* Tilia spp.*,* Sophora japonica*, and* propolis* [60].

### 2.3. Myricetin

Myricetin (3,3′,4′,5,5′,7-hexahydroxyflavone) is a natural flavonol found in vegetables, fruits, berries, tea, and medicinal plants [70]. Various health related studies on myricetin from plant sources have been demonstrated which revealed the antioxidant, antiviral, anticarcinogenic, antiplatelet, hypoglycemic, and cytoprotective properties of myricetin [59, 71–76]. Myricetin also possesses antihypertensive action. Godse et al. [77] reported that myricetin prevent the progression of high blood pressure and reversed the metabolic alterations in fructose-induced rats. Besides, myricetin was proven to suppress body weight gain and fat accumulation by increasing oxidation of fatty acids which is due to upregulation of hepatic peroxisome proliferator activated receptor (PPAR*α*) and downregulation of hepatic sterol regulatory element-binding proteins (SREBPs) expressions in high fat-induced rats. These results revealed the antiobesity and antihyperlipidemic effects of myricetin [78]. Myricetin also was proven to possess protective effects on the oxidation of LDL in blood [79, 80]. Ha et al. [79] reported that* Ampelopsis cantoniensis* has myricetin as its main constituent and managed to inhibit the LDL oxidation induced by metal ion (Cu^2+^) and free radical (AAPH), and therefore the* A. cantoniensis* extract can be utilized as a natural remedy to prevent the oxidation of LDL which is involved in the formation atherosclerotic lesion. Lian et al. [80] revealed that besides preventing the LDL from oxidation, myricetin also blocks the oxidized LDL uptake by macrophages and plays an essential role in preventing atherosclerosis.* In vivo* studies on antiartherosclerotic effects of myricetin could further provide better knowledge and understanding of its role in ameliorating atherosclerosis. High content of myricetin has been also reported in these plants:* Myrica cerifera* L [81],* Calamus scipionum* [54],* Chrysobalanus icaco* L [82],* Moringa oleifera*, and* Aloe vera* [17].

### 2.4. Rutin

Rutin (quercetin-3-rutinoside) is a bioflavonoid commonly found in buckwheat bran, black tea, and citrus fruits [83]. Rutin contributes to many positive health effects such as powerful antioxidant [84], protects against free radicals [85], possess anti-inflammatory properties [86, 87], and suppresses aldose reductase activity [88]. Endothelial dysfunction plays a major role in the development of CDVs and it is found in conditions such as hypertension, hypercholesterolemia, and atherosclerosis. Rutin in buckwheat extract decreases body weight, improves capillary fragility to maintain blood pressure, and significantly reduced nitrotyrosine immunoreactivity in endothelial cells of aorta [89]. Rutin has been proven to exhibit antiobesity effect via suppression of oxidative stress, dyslipidemia, and hepatosteatosis in obese rats. Rutin decreases liver and adipose tissue weight, suppresses hepatic triacylglycerol and cholesterol level, and enhances antioxidant enzymes (superoxide dismutase and glutathione peroxidase) activities in obese rats [90]. On the other hand, rutin plays a great role in preventing atherosclerosis and the evidence for its antiatherosclerotic effects is available in* in vivo* studies: rabbits [91], rat [92], and hamsters [93]. Voskresensky and Bobyrev [91] showed that rutin delays the hypercholesterolemia development and inhibits the atherosclerotic formation in rabbits' aorta. While, Santos et al. [92] researched into the effects of rutin on controlling lipid metabolism and found that rutin reduced the cholesterol levels and has the lowest level of triacylglycerol in hypercholesterolemia rats. In addition, rutin extracted from* Dimorphandra mollis* showed decreases in the level of plasma triglyceride of hypercholesterolemia induced hamsters without changing the high-density lipoprotein (HDL) cholesterol and total cholesterol levels. Rutin was also proven to be nontoxic and no notable changes were observed in total white bloods cells and mononuclear and granulocytes cells compared to the untreated control group [93]. Therefore, rutin can be developed as an alternative drug for the treatment of atherosclerosis. Other plants that constitute rutin compound are* Flos hippocastani* [94],* Ruta graveolens* [95],* Rhus cotinus* [96], and* Phyllanthus amarus* [97].

### 2.5. Naringenin

Naringenin (4′,5,7-trihydroxyflavanone) has been widely studied in issues related to atherosclerosis. Naringenin was reported to have poor antioxidant properties compared to other flavanoids but it is still able to be a potential inhibitor in cholesterol biosynthesis [98, 99]. Borradaile et al. [100] claimed that naringenin regulates apolipoprotein B secretion by HepG2 cells directly through inhibition of cholesterol ester synthesis. Naringenin also reported to affect lipid metabolism through inhibition of acyl coenzyme A: cholesterol O-acyltransferase and 3-hydroxy-3-methylglutaryl-coenzyme A (HMG CoA) reductase in rats [101, 102]. Study on higher animal models, rabbits, was conducted by Kurowska et al. [103] whereby the results obtained showed decrease in hepatic cholesterol and LDL levels. Meanwhile, studies performed on hypercholesterolemic human subjects showed increase in HDL levels after consumption of naringenin rich orange juices [104]. Lee et al. [105] demonstrated that (2S)-naringenin, isolated from* Typha angustata*, inhibits the vascular smooth muscle cells (VSMCs) proliferation while Mulvihill et al. [106] reported that naringenin-treated mice showed about 60% reduction of aortic cholesterol and decreases the level of hepatic cholesteryl ester, very low-density lipoprotein (VLDL) and low density lipoprotein (LDL). These lead to the reduction in cholesterol and triglyceride accumulation within the arterial wall and ameliorates atherosclerosis. Besides, naringenin also prevents accumulation of adipose, adipocyte hypertrophy, and dyslipidemia [106]. Other biological activities of naringenin include anti-inflammatory, anticancer, and positive effects on sex metabolism through binding to estrogen receptors [107–112]. Naringenin is a potential flavonoid to be explored further especially in atherosclerosis since it has numerous health benefits. Some of the plant sources that are rich in naringenin are* Solanum lycopersicum* and citrus fruits [113, 114],* Mentha aquatica* L [115], immature fruit of* Citrus aurantium* [116], and flowers of* Acacia podalyriifolia* [117].

### 2.6. Catechin

Catechin [(2R,3S)-3′,4′,5,7-tetrahydroxyflavan-3-ol] has been reported to effectively inhibit lipid peroxidation and scavenge free radicals [118, 119]. Catechin is known to possess preventive effect in CVDs due to its involvement in oxidative process in atherogenesis [120]. Being antioxidant, catechin is able to modulate cellular signaling pathways that lead to elevation of vascular reactivity, platelet aggregation, and reduction of inflammation [121–124]. Diverse studies have been conducted using tea (*Camellia sinensis*) which contains catechin believed to play a major role as cardioprotective plant source [123]. Almost 50–80% of the total catechin from tea is epigallocatechin-3-gallate (EGCG) and it is considered to be the most effective bioactive component in cholesterol lowering [125]. Proinflammatory cytokine and tumor necrosis factor-alpha (TNF*α*) commonly exist in atherosclerotic lesions which have direct effect on monocyte chemotactic protein-1 (MCP-1) stimulation and vascular endothelial cells. MCP-1 plays an important role in the monocytes' recruitment in developing inflammatory CDVs. EGCG inhibits TNF*α* activation and resulted in reduction of MCP-1 production in coronary vascular endothelial cell [126]. Furuyashiki et al. [127] demonstrated that EGCG at low concentration (5 *μ*M) is able to suppress intracellular lipid accumulation in an* in vitro* model suggesting that the cholesterol lowering effect of EGCG is due to the influence on intestinal lipid absorption. Clinical studies done by Potenza et al. [128] in hypertensive rats suggested that EGCG was shown to reduce blood pressure, raise adiponectin levels, protect against myocardial injury, and improve endothelial function which was proven to reduce the CVDs risk. Another study done by Bursill and Roach [129] confirmed that EGCG lowers the cholesterol and triglyceride absorption in rats. Studies conducted on humans by Widlansky et al. [130] claim that EGCG can reverse endothelial dysfunction and improve dilation of brachial artery in patients with coronary artery disease. These studies suggest the efficiency of EGCG as a potential agent for treating CVD. Other plant sources that are rich in catechins are* Betula pubescens* and* Betula pendula* [131],* Cocos nucifera* [132], fruit pulp of* Argania spinosa* [133], and* Cassia fistula* [134].

### 2.7. Fisetin

Fisetin (3,7,3′,4′-tetrahydroxyflavone) together with morin and myricetin is structural related flavan-3-ol and is commonly distributed in vegetables and fruits such as apple, strawberry, grape, persimmon, cucumber, and onion at concentrations of 2–160 *μ*g/g [135]. Fisetin is known for its strong antioxidative [136], anti-inflammatory [137], anticancer [138], antiproliferative [139], and antihyperglycemic [140] activities. Increase in adipocyte cell number (hyperplasia) is an essential therapeutic target for the prevention of obesity [141]. Jung et al. [142] demonstrated that fisetin ameliorates diet-induced obesity by inhibition of mammalian target of rapamycin complex I (mTORCI) signalling which is central mediator for lipid biosynthesis, cellular growth, and proliferation. Fisetin supplementation in high-fat diet-induced mice regulated fat accumulation in adipose tissue and suppresses adipogenesis during the adipocyte differentiation via downregulation of related gene and thus the study proves that fisetin can be an effective antiobesity agent [142]. The development of atherosclerotic lesion is induced by the elevated concentrations of LDL, blood cholesterol, and triglycerides [143]. Macrophages play an essential role in the development of atherosclerosis by accumulating cholesterol in foam cells [144]. Fisetin inhibits LDL oxidation by macrophages and plays a role as free radical scavenger in LDL which also inhibits the oxidative enzymes from macrophage [145]. Thiobarbituric acid-reactive substances assay (TBARS), electrophoretic mobility, and conjugated diene formation analyses by Lian et al. [80] have shown that fisetin inhibits Cu^2+^ mediated LDL oxidation stronger than morin and myricetin. Binding of CD36 (class B scavenger receptor) to oxidized LDL causes the formation of atherosclerotic lesion. Fisetin blocks macrophage's oxidized LDL uptake by reducing the CD36 expression on the macrophages [80]. However, the study of fisetin* in vivo* atherosclerosis models is still lacking. The participation of fisetin in ameliorating atherosclerosis can further be confirmed in animal model studies for future flavonoids-based drugs. Plants like* Butea frondosa*,* Gleditsia triacanthos*,* Quebracho colorado* [146],* Curcuma longa* [147],* Rhus verniciflua* [148],* Acacia greggii*, and* Acacia berlandieri *[149] are rich sources of fisetin.

### 2.8. Gossypetin

Gossypetin (3,5,7,8,3′,4′-hexahydroxyflavone) was originally isolated from* Hibiscus spp. *[151]. Gossypetin suppressed the oxidation of LDL [143] and was able to modify the LDL in a form accepted by macrophage through elevated affinity process in a nonoxidative mechanism [154]. Lin et al. [150] reported that gossypetin is an important flavonoid from* Hibiscus sabdariffa* and has been shown to prevent atherosclerosis, reduce oxidative stress, and neutralize agents that cause cancer.* H. Sabdariffa *extract revealed the potential of gossypetin in inhibiting atherosclerosis in hyperlipidemic rabbits [150]. Chen et al. [151] published the first report on the antiatherosclerotic activity of gossypetin in* in vitro* study and demonstrated that gossypetin inhibits both lipoprotein oxidation and lipid peroxidation. Gossypetin functions against oxidative LDL and accumulation of intracellular lipid through the regulation of peroxisome proliferator-activated receptor (PPAR) signals which stimulated the cholesterol to be removed from macrophages and retard the atherosclerosis process [151]. The findings mentioned strongly suggest the development of gossypetin as an antiatherosclerotic agent.* H. Sabdariffa* extract has been used as antihypertensive agent since it decreases systolic blood and pulse pressure [155]. Villalpando-Arteaga et al. [156] reported that* H. Sabdariffa* aqueous extract possesses antilipidemic, antiobesity, and hepatoprotective effects. Studies on the role of gossypetin against hypertension and obesity can further reveal its beneficial effects and pharmacological activities. Besides* H. Sabdariffa*, gossypetin also is present in* H. vitifolius*,* H. esculentus*,* Empetrum nigrum* and* Acacia constricta* [152],* H. rosa-sinensis*,* Chiranthodendron pentadactylon*,* Fremontia californica*,* Thespesia populnea*, and* Fagonia cretica* [153].

## 3. Conclusion

The summary of reported plant flavonoids is shown in Table 1. Various flavonoids compounds that are available in plants exhibit numerous effects that can prevent the progression of atherosclerosis and diseases such as hypercholesterolemia, hypertension, and obesity that can lead to CVDs.* In vivo* studies on myricetin and fisetin could give better view on its potential as an antiatherosclerotic agent. Investigation of potential effects of gossypetin against hypertension and obesity is suggested. Future research can be focused on the role of plant flavonoids in human metabolism and signaling pathway involved during the therapy of atherosclerosis. This could help to determine the strategies of improving the alternatives therapeutic approaches for atherosclerosis and other related diseases. Since there is an urge for alternatives natural treatment due to the side effects of statins, flavonoid-based drugs can be utilized for the prevention and treatment of atherosclerosis and CVDs.

## Figures and Tables

**Table 1:** Summary of reported plant flavanoids.

Flavonoid	Bioavailability	Plant
Quercetin	Anti-inflammatory [35] Antihypertensive [40] Vasodilator effects [41] Antiobesity [47] Antihypercholesterolemic and antiatherosclerotic [50]	*Morus alba *L [48] *Camellia chinensis* [53, 54] *Allium fistulosum *and* Calamus scipionum*[54] *Moringa oleifera* [17, 55] *Centella asiatica* [56] *Hypericum hircinum* [57] *Hypericum perforatum* [58]

Kaempferol	Enhances endothelium vasorelaxation [61] Protective effects against endothelial damage [62] Reduce oxidative stress [63] Antiatherosclerotic [65] Antihyperlipidemic [67]	*Moringa oleifera* [17, 55] *Centella asiatica* [56] *Ginkgo biloba, Equisetum spp., Tilia spp., Sophora japonica,* and *propolis* [60] *Euonymus alatus* [68] *Kaempferia galanga* L [69]

Myricetin	Antiplatelet [75] Cytoprotective effects [76] Antihypertensive [77] Antiobesity and antihyperlipidemic [78] Antiartherosclerotic [80]	*Calamus scipronum* [54] *Moringa oleifera* and *Aloe vera* [17] *Ampelopsis cantoniensis* [79] *Myrica cerifera* L [81] *Chrysobalanus icaco* L [82]

Rutin	Anti-inflammatory [86, 87] Improves capillary fragility and antihypertensive [89] Suppresses oxidative stress and antiobesity [90] Antiartherosclerotic [91] Antihypercholesterolemic [93]	*Dimorphandra mollis* [93] *Flos hippocastani* [94] *Ruta graveolens* [95] *Rhus cotinus* [96] *Phyllanthus amarus* [97]

Naringenin	Antihypercholesterolemic [104] Antiatherogenic and antiobesity [106] Anti-inflammatory [111]	*Typha angustata* [105] *Solanum lycopersicum* and *citrus fruits* [113, 114] *Mentha aquatica* L [115] *Citrus aurantium* [116] *Acacia podalyriifolia* [117]

Catechin	Antiplatelet and anti-inflammatory [121–124] Cardioprotective effects [123] Antiatherosclerotic [126] Antihypercholesterolemic [127] Antihypertensive [128]	*Camellia sinensis* [123, 125–130] *Betula pubescens and Betula pendula* [131] *Cocos nucifera* [132] *Argania spinosa* [133] *Cassia fistula* [134]

Fisetin	Antioxidative [136] Anti-inflammatory [137] Antiproliferative [139] Antiobesity [142] Antiatherosclerotic [80]	*Butea frondosa, Gleditsia triacanthos, *and* Quebracho colorado* [146] *Curcuma longa* [147] *Rhus verniciflua* [148] *Acacia greggii* and *Acacia berlandieri* [149]

Gossypetin	Suppresses LDL oxidation [143] Reduces oxidative stress [150] Antihyperlipidemic [150] Antiatherosclerotic [151]	*Hibiscus spp*. [151] *Hibiscus sabdariffa* [150] *Hibiscus vitifolius, Hibiscus esculentus, Empetrum nigrum,* and *Acacia constricta* [152] *Hibiscus rosa-sinensis, Chiranthodendron pentadactylon, Fremontia californica, Thespesia populnea, and Fagonia cretica* [153]
